# Level of Patient Satisfaction with Quality of Primary Healthcare in Almaty During COVID-19 Pandemic

**DOI:** 10.3390/ijerph22050804

**Published:** 2025-05-21

**Authors:** Dinara Shaki, Gulshara Aimbetova, Venera Baysugurova, Marina Kanushina, Aigerim Chegebayeva, Muratkhan Arailym, Erkebulan Merkibekov, Indira Karibayeva

**Affiliations:** 1 School of Public Health, Asfendiyarov Kazakh National Medical University, Almaty 050000, Kazakhstan; dnrshaki@gmail.com (D.S.); vbaisugurova@mail.ru (V.B.); 2 Faculty of Postgraduate Studies, AC Institute of International Education, Prague 10200, Czech Republic; marinakan46@gmail.com; 3 Veteran Clinic Almaty, Almaty 050000, Kazakhstan; baideshova7@mail.ru; 4 Faculty of Medicine and Health, Al-Farabi Kazakh National University, Almaty 050040, Kazakhstan; muratkhan.arailym@med-kaznu.com; 5 Multiprofile City Hospital, Taraz 060000, Kazakhstan; merkibekov@gmail.com; 6 Department of Health Policy and Community Health, Jiann-Ping Hsu College of Public Health, Georgia Southern University, Statesboro, GA 30458, USA; ik01379@georgiasouthern.edu

**Keywords:** primary healthcare, COVID-19, pandemic, patients’ satisfaction, Kazakhstan

## Abstract

Background: This study aimed to assess patient satisfaction with the quality of healthcare services at selected public primary healthcare facilities in Almaty during the COVID-19 pandemic and to identify associated demographic and facility-related factors. Methods: A cross-sectional, quantitative study was conducted over a period of 6 months, from 30 June to 31 December 2021, through a web-based survey. An adapted questionnaire was employed to survey the respondents. In total, 1035 respondents participated in the study. To examine the relationship between demographic and facility characteristics and patient satisfaction, we utilized the proportional odds model for ordinal logistic regression. Results: A total of eight primary healthcare organizations from the public sector in Almaty participated in the survey. The analysis identified significant demographic predictors of patient satisfaction, such as marital status, social status, self-perceived health, and the use of online consultations. Among the facility-related factors, only the availability of a cross-ventilation system emerged as a significant predictor. Conclusions: This study provides evidence for the factors influencing patient satisfaction with primary healthcare services in Almaty during the COVID-19 pandemic. Both demographic characteristics and facility-level attributes were found to significantly affect satisfaction levels. These findings underscore the need for targeted structural and organizational improvements in primary healthcare settings, especially during public health emergencies. Addressing these gaps through infrastructural upgrades, enhanced preparedness, and the integration of patient-centered care models can help to bolster trust and resilience within Kazakhstan’s healthcare system.

## 1. Introduction

The COVID-19 pandemic presented an unprecedented challenge to health systems worldwide, impacting the quality and availability of healthcare delivery [1]. During the coronavirus pandemic, health systems around the world, including primary healthcare services, were challenged by an increasing demand for care for people with COVID-19. The situation was further exacerbated by fear, stigma, misinformation, and travel restrictions that disrupted the provision of care for all conditions. Overwhelmed health systems led to delays in the provision of care to people in need, resulting in sharp increases in both direct mortality from the outbreak and indirect mortality from preventable and treatable conditions [2,3,4].

Due to the lack of vaccines and specific drugs and the high contagiousness of the infection, the only effective measures to combat the pandemic were public health measures such as isolation, social distancing, and case monitoring to reduce transmission and slow the pace of the pandemic. It was also necessary to equip the health system with resources to provide adequate and timely care to people with COVID-19 [5].

The Republic of Kazakhstan, like many other countries, faced the challenges presented by the COVID-19 pandemic, leading to the need to assess and optimize the effectiveness of PHC. The first COVID-19 case in Kazakhstan was diagnosed on 13 March 2020 [6]. The government imposed quarantine measures in all regions starting on 22 March 2020, locking down major cities such as Almaty, Nur-Sultan, and Shymkent. All hospitals across the country were forced to accept infected patients and those with regional contacts, as well as open additional hospitals, beds, and expand their capacity to effectively deal with the epidemic situation. Treatment protocols were developed based on international experience and guidelines. The government imposed strict travel restrictions on cities and regions—all air and ground transportation was suspended, including restrictions on entry and exit from major cities, and all citizens were required to self-quarantine and stay at home [7].

COVID-19 call centers were implemented, and citizens of the Republic of Kazakhstan could call 24 h a day to find out all the information about the current situation regarding the incidence of coronavirus, as well as about preventive measures and the main symptoms of the disease. These centers were established as part of city ambulance stations, primary healthcare facilities, and the emergency departments of medical organizations that had operational communication with the Internal Affairs and civil protection services. Consultations at call centers were provided by consulting doctors, senior doctors from city emergency medical services, experienced paramedics, and other healthcare professionals. The call center specialists informed the population about the provision of medical care at the pre-hospital stage and answered frequently asked questions related to coronavirus infection. In the event of symptoms such as headache, runny nose, chest pain, fever, tachycardia, or rapid breathing, calls were redirected to dispatcher 103 [8].

In 2020, a total of 16 modular infectious disease hospitals were built and put into operation in Kazakhstani cities, 44 outpatient care facilities were reconstructed, including 30 facilities in rural areas, 3 infectious disease hospitals, and 64 oxygen stations, and 63 outpatient clinics were put into operation. Measures were taken to provide the population with medicines, medical devices, and personal protective equipment [9].

As a measure to combat coronavirus infection, mass vaccination of the population began. Vaccination against COVID-19 was carried out in Kazakhstan starting on 1 February 2021, and 55.35% of the population had received full vaccination by the end of 2021 [10]. The domestic vaccine QazVac (Kazakhstan) was used, as well as Sputnik V (Moscow, Russia), Sinopharm (Beijing, China), Sinovac (Beijing, China), and Pfizer (New York, NY, USA) [11]. Primary healthcare providers play a key role in informing people about vaccines, encouraging them to vaccinate, and keeping vaccine coverage high in their populations [12,13,14].

One of the main tasks of the Kazakhstani government is to provide the population with accessible and high-quality medical care. A high-quality healthcare system is impossible without a strong primary healthcare system. Strengthening primary healthcare can not only reduce the impact of COVID-19 on the health and well-being of the population, but also increase resilience to the next possible epidemic disease [15].

Primary healthcare played a significant role during the pandemic, contributing to the early detection, diagnosis, treatment, and referral of people with COVID-19 [16]. The World Health Organization (WHO) has stressed the role of primary care as the most efficient and effective strategy to improve the health of populations [17]. Primary healthcare serves as a gatekeeper in a country’s healthcare system, emphasizing health promotion, disease prevention, treatment, rehabilitation, and palliative care [18]. Strong and resilient primary healthcare systems are designed to predict, prevent, prepare, absorb, adapt, and transform when faced with crises, ensuring the continuity of routine health services [19]. During a pandemic, it is extremely important for people to have access to health services for both emergency and primary healthcare. The pandemic brought to light the vulnerabilities of PHC systems, noting a significant decrease in the use of primary care services for non-emergency conditions. Routine health services, including immunizations, prenatal care, and chronic disease management, were severely impacted [20].

The Alma-Ata Declaration and the Astana Declaration on Primary Health Care in 2018 emphasize the importance of primary healthcare for universal healthcare coverage and the Sustainable Development Goals agenda until 2030 [21]. Kazakhstan is undergoing comprehensive healthcare reform aimed at restructuring its public healthcare system and improving primary healthcare services [22]. Improving access to medications, interpersonal communication skills, and technical assistance are three main priorities for enhancing the perceived quality of primary healthcare and health policy measures [23].

Since 1 January 2020, Kazakhstan has been implementing compulsory social health insurance [24]. The pandemic has certainly highlighted the importance of compulsory social health insurance, as many people diagnosed with COVID-19 had access to a wide range of medical services covered by compulsory social health insurance, such as free testing for COVID-19 (PCR tests), rehabilitation after infection, reduced personal expenses, access to expensive services, priority access to services, and social support in the form of maintaining an insured status after losing a job during the crisis.

Healthcare in Kazakhstan has been undergoing active reform to achieve international healthcare standards since the country’s independence. Recent publications have highlighted that both patient experience and patient satisfaction are integral components of a high-quality health system framework and targets for improvement in the transition to high-quality health systems in low-income and middle-income countries [25]. Patient satisfaction is the degree to which patients are pleased with their healthcare, both inside and outside healthcare facilities [26]. Little is known about patient satisfaction with primary care providers during the pandemic, when people had little choice but to seek remote care [27]. Therefore, patient satisfaction is considered as an important measure to evaluate the quality of health services and can predict both compliance and utilization [28]. Assessing patient satisfaction with healthcare during a state of emergency provides useful lessons for effective responses.

Given the importance of understanding the patient experience during the COVID-19 pandemic, this study aimed to assess patient satisfaction with primary healthcare services in Almaty city and identify associated demographic and facility-related factors. To solve this problem, a specific question was asked, as follows: what factors influenced patient satisfaction with primary healthcare services in Almaty during the COVID-19 pandemic? Patient satisfaction research is very important, especially in emergency situations such as the COVID-19 pandemic, as it helps to identify gaps that occurred during the emergency and learn useful lessons for the future, also helping to improve primary healthcare systems.

## 2. Materials and Methods

### 2.1. Study Design and Settings

This cross-sectional study was conducted at 8 primary healthcare service centers in Almaty city, Kazakhstan, in 2021. Participants were recruited from 30 June to 31 December 2021. Data were collected through a web-based survey.

In this study, an online questionnaire was used as the primary tool for data collection. The questionnaire consisted of closed-type questions and was designed to collect quantitative data, which allowed for statistical analyses of the respondents’ feedback.

The questionnaire consisted of the following two parts: the sociodemographic characteristics of the patients and facility characteristics. The first part included items on sociodemographic factors and self-reported health. These items were age, gender, marital status, social status, and region of residence within the city of Almaty (this question was included because quality of life can vary significantly across different regions of the city). Other questions in this section assessed self-reported health status, the presence of chronic conditions, participation in regular health screenings, and patient satisfaction with online consultations. The second part of the questionnaire included questions on the characteristics of the facility, such as the presence of separate entry and exit doors, the existence of cross-ventilation systems, the availability of hand washing and sanitizing points, the availability of face masks, the implementation of preventive measures, and the evaluation of sanitary and hygienic conditions.

The development of the questionnaire involved several key stages, starting with the definition of the research objectives and the formulation of the questions, which were derived from the studies by Suneela Garg et al. [29] and V.N. Buzin et al. [30]. Elements from these tools were adapted after reaching an expert consensus to ensure relevance to the study’s context and objectives while maintaining methodological rigor. A pilot test of the survey was conducted with a small sample of participants (*n* = 20) to evaluate its content validity, ensuring that the questions accurately reflected the constructs being measured. These questions were aimed at reflecting various aspects of patient satisfaction. To assess reliability, internal consistency was tested using Cronbach’s alpha coefficient, which yielded a value of 0.79. After adjustments, the questionnaire was validated using statistical methods, including factor analysis to verify the structure of the scales. Final testing of the questionnaire on a larger sample confirmed its reliability and measurement accuracy.

### 2.2. Source and Study Population

The study population consisted of all patients who were enrolled in the 8 primary healthcare organizations we selected and who had attended a visit with a primary healthcare physician between 30 June and 31 December 2021.

In total, 1350 respondents took part in the survey and provided valid responses, defined as answering at least 80% of the questions in the questionnaire. However, since not all respondents answered the question, ‘Overall, are you satisfied with the quality of primary healthcare provided during the epidemic?’, we analyzed the answers of 1035 respondents who provided valid responses to this specific question, while the remaining responses were treated as missing data. The 1350 valid responses represented a 78% response rate. There were no instances of non-response to any specific question in the survey.

We calculated in OpenEpi The sample size was calculated using the following estimation formula: *n* = [DEFF × Np(1 − *p*)]/[(d^2^/Z^2^_1−α/2_×(N − 1) + *p*×(1 − *p*)]

Z1 − α = 1.96 (95% confidence interval)

Population proportion P = 57.7% [29]

D = 0.05 (5% absolute precision)

N = 761 proposed

N = 1730 final (including 44.1% non-response rate [30])

### 2.3. Procedure

We collected data through a rapid survey using a pretested Google Forms online digital questionnaire. A random sampling method was used to select the study participants. In total, 50 public primary healthcare organizations were selected, from which we chose 8 organizations from the existing 8 regions of the city of Almaty using the convenience sample method. A patient database with phone numbers was compiled from the patient registries of the primary healthcare organizations who agreed to be contacted about the healthcare surveys, which were obtained with the permission of the administration. These patients were sent an invitation to participate in the study through email, WhatsApp, Telegram, and instant messages containing the Google Form survey link. Before taking part in the study, participants were required to provide electronic informed consent, verifying that they were at least 18 years old and agreed to participate voluntarily.

### 2.4. Inclusion and Exclusion Criteria

All patients over 18 years of age and registered at the selected primary healthcare organizations were included in the study. Patients who were unable to respond due to health reasons were excluded from the study.

### 2.5. Study Variables

The dependent variable in this study was patient satisfaction, assessed through the question “Overall, are you satisfied with the quality of primary healthcare provided during the epidemic?” with the following response options: (1) Difficult to answer; (2) Not satisfied; (3) Rather dissatisfied than dissatisfied; (4) Not fully satisfied; (5) Rather satisfied than satisfied; and (6) Satisfied. A proportional odds model was employed for the analysis, treating patient satisfaction as an ordinal variable arranged in ascending order to reflect increasing levels of satisfaction.

The independent variables of the present analysis were divided into the following two groups: patient demographic characteristics and primary healthcare facility characteristics. Demographic characteristics included age, gender, marital status, social status, Almaty region of residence, self-described health status, chronic disease, dispanserization, and online consultation. Facility characteristics included entry and exit, cross-ventilation system, hand washing/sanitizing points, face mask availability, prophylactic measures, and assessments of sanitary and hygienic conditions.

### 2.6. Statistical Analysis

Statistical analyses were performed using R (version 4.3.2). Categorical variables are summarized as frequencies and percentages, while continuous variables are presented as means and standard deviations. To examine the relationship between demographic and facility characteristics and patient satisfaction, we utilized the proportional odds model for ordinal logistic regression. For each predictor, adjusted odds ratios (AORs) and 95% confidence intervals (CIs) were calculated. Statistical significance was determined at *p* < 0.003, based on a Bonferroni correction applied to account for multiple comparisons across the independent variables included in the analysis. Respondents with missing data (*n* = 315) were excluded from the analysis to ensure data integrity. We utilized the listwise deletion method, ensuring that respondents with incomplete responses were excluded from the analysis. This approach was implemented to maintain the integrity and reliability of the dataset, minimizing potential biases caused by missing data.

The Local Ethics Committee of Asfendiyarov Kazakh National Medical University approved this study, protocol No. 8, dated 30 June 2021. Written informed consent was obtained from the participants at the time of questionnaire completion and was attached to the questionnaire.

## 3. Results

### 3.1. Participant Demographics

A full description of the patient demographic and facility-related characteristics is presented in Table 1. In terms of age, respondents who chose the options “Satisfied” (36.5 ± 14.3), “Rather satisfied than dissatisfied” (31.2 ± 11.8), and “Not fully satisfied” (36.5 ± 13.1) were generally younger than those who selected “Rather dissatisfied than satisfied” (37.0 ± 13.6), “Not satisfied” (42.4 ± 13.2), and “Difficult to answer” (39.8 ± 12.7). The study included 632 women (61%) and 403 men (39%). The distribution of satisfaction levels with the quality of medical care was as follows: 45.2% of respondents were satisfied, of whom 28.4% were women; 12.7% were rather satisfied than dissatisfied, with women comprising 7.3%; 16.9% were not fully satisfied, including 10.0% women; 7.0% were rather dissatisfied than satisfied, of whom 4.3% were women; 7.2% were not satisfied, including 4.7% women; and 11.0% found it difficult to answer, with 6.4% being women.

### 3.2. Demographic Predictors of Patients’ Satisfaction with the Quality of Primary Healthcare

**Figure 1:**
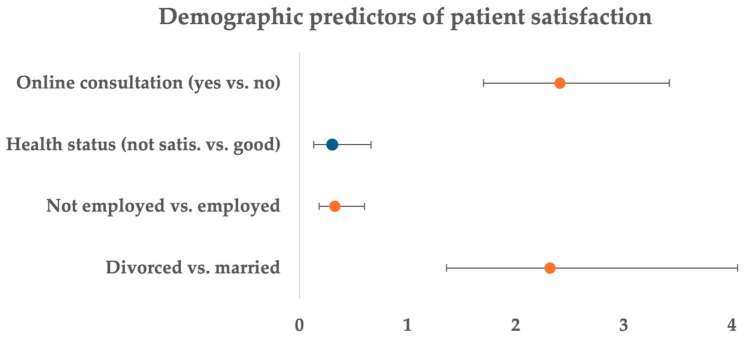
Demographic predictors of patient satisfaction.

Table 2 and Figure 1 present the results of the ordinal logistic regression analysis, identifying the significant factors associated with patient satisfaction regarding the quality of primary healthcare services. Among the demographic characteristics, marital status, social status, self-reported health status, and the use of online consultations were significant predictors of satisfaction. Specifically, divorced respondents had 2.32 times higher odds of being more satisfied with the quality of care compared to married respondents (AOR = 2.32, 95% CI [1.36–4.05]). Respondents who were unemployed had significantly lower odds of being satisfied than those who were employed (AOR = 0.33, 95% CI [0.18–0.60]). Similarly, respondents who rated their health status as “not satisfactory” had lower odds of reporting satisfaction with care compared to those who rated their health as “good” (AOR = 0.30, 95% CI [0.13–0.66]). Lastly, patients who accessed care through online consultations had significantly higher odds of satisfaction compared to those who did not (AOR = 2.41, 95% CI [1.70–3.42]).

Among the facility attributes, the availability of a cross-ventilation system was a significant predictor of patient satisfaction. The odds of a patient being satisfied with the quality of care were significantly higher in healthcare facilities with cross-ventilation systems compared to those without (AOR = 1.96, 95% CI [1.37–2.79]) (Table 3).

## 4. Discussion

This study examines the influence of patient demographic and healthcare facility characteristics on overall patient satisfaction with the quality of primary healthcare. The analysis identifies significant demographic predictors of patient satisfaction, such as marital status, social status, self-perceived health, and the use of online consultations. Among the facility-related factors, only the availability of a cross-ventilation system emerges as a significant predictor.

The findings of our analysis are consistent with previous research highlighting the influence of demographic factors on patient experiences during the pandemic [31]. A study conducted in Greece also showed a strong negative association between loss of employment and patient satisfaction [32]. Respondents who rated their health status as “not satisfactory” had lower odds of reporting satisfaction with care compared to those who rated their health as “good”. This finding corroborates study findings from a Midwestern metropolitan area of the United States, which found higher patient satisfaction among individuals with better self-rated health (*p* < 0.001) [19]. The lower satisfaction among our study’s participants with chronic diseases may be linked to the introduction of compulsory social health insurance in Kazakhstan on 1 January 2020, which coincided with the COVID-19 outbreak two months later. This system aimed to reform healthcare financing and enhance the accessibility and quality of medical services [22].

Online consultation has several advantages and disadvantages compared to in-person visits, which could alter patient satisfaction with the delivered care. Telemedicine has the ability to reduce the amount of time needed to deliver medical care, increase patient convenience, and decrease the risk of exposure to infectious agents, such as COVID-19 [33]. In our study, online consultation was a significant predictor of patient satisfaction. In a similar study conducted in China, out of 2599 pregnant women, a total of 957 participants completed the satisfaction part of the survey, and 94.63% of these participants were completely or mostly satisfied with online consultations [21]. The widespread adoption of telemedicine technologies, including video consultations, was one of the most significant transformations in healthcare during the COVID-19 pandemic. The level of digitalization in Kazakhstan’s healthcare system plays a crucial role in shaping patient experiences with online consultations. While younger populations may be more familiar and comfortable with digital technologies, older patients may face challenges related to digital literacy and access, which could impact their satisfaction [34]. A useful lesson in telemedicine can be learned from the practices carried out in Uzbekistan and Kyrgyzstan, where students from local medical universities were involved in conducting preventive consultations with local populations [35,36].

Evidence also suggests that age, trait anxiety, and perceived COVID-19 threat significantly affected well-being and life satisfaction during the pandemic [37]. During the COVID-19 pandemic, patients’ views on the quality of medical care changed significantly. A number of restrictions associated with the pandemic led to a decrease in the level of patient satisfaction [26,27]. This may be attributed to the fact that healthcare systems in low- and middle-income countries are often inadequately prepared to manage chronic conditions such as hypertension, diabetes, and other non-communicable diseases during emergencies like pandemics. These systems are typically better equipped to address acute infectious and parasitic diseases [38]. Psychological factors also influence patients’ perceptions of medical care. It is well-established that psychological factors play a crucial role in adherence to public health measures, such as vaccination, and in individuals’ ability to cope with the threat of infection and its consequences. These factors are particularly important in the management of infectious diseases, including COVID-19. Psychological reactions to pandemics often manifest as maladaptive behaviors, emotional distress, and defensive responses, with individuals prone to psychological difficulties being especially vulnerable [29]. This is supported by a study conducted in the United States in June 2020, where elevated levels of adverse mental health conditions, substance use, and suicidal ideation were reported among adults. The prevalence of anxiety disorder symptoms was approximately three times higher than that reported in the second quarter of 2019 (25.5% versus 8.1%), and the prevalence of depressive disorder was approximately four times higher than that reported in the second quarter of 2019 (24.3% versus 6.5%) [39]. However, technologies such as telemedicine have improved the availability of health services, which has increased satisfaction among certain groups of patients [40,41].

Our findings indicate that the odds of patients being satisfied with the quality of care were significantly higher in healthcare facilities equipped with cross-ventilation systems. Such systems help to prevent the spread of pathogenic microbes, thereby safeguarding the health of both staff and patients [7]. Cross-ventilation is linked to patient satisfaction because it improves air quality, temperature control, and lighting, which creates a more comfortable and pleasant environment [42]. Fresh air and natural light contribute to a better mood and well-being, while reduced noise levels and a perception of cleanliness enhance the overall patient experience [43]. A noticeable share of 4.1% of global deaths in recent decades have been caused by severely poor indoor air quality, especially nosocomial infections [44]. In our study, the likelihood of a patient not being satisfied was high when cross-ventilation was not in place.

It is surprising that, in our study, the likelihood of patient satisfaction with the quality of care was not significantly associated with the shortage of face masks in primary care settings. It is known that during the outbreak of coronavirus infection, there was a shortage of personal protective equipment. Health experts called on the federal government to take steps to mobilize and distribute adequate supplies of protective equipment, especially gloves, medical masks, goggles or face shields, gowns, and respirators. Respirators, which have been shown to be effective in reducing respiratory infections among healthcare workers, were in particularly short supply [45]. Due to the critical shortage of masks, WHO also suggested washing cloth masks in soap or detergent and then preferably soaking them in hot water of at least 60 °C for 30 min [46]. Similar problems occurred in neighboring Uzbekistan, such as shortages of beds, medical supplies, and personal protective equipment (PPE) for health workers [47]. Also, a similar picture related to coronavirus infection can be seen from the experience of Kyrgyzstan, whose healthcare system at that time can be described by the following two aspects: half of artificial lung ventilators did not work, and every fourth registered COVID-19 case was among healthcare workers [48]. Moreover, the overloaded healthcare system forced service providers to focus on fighting the pandemic, which led to limited access to medical care for patients suffering from other diseases [49].

A central strategy for healthcare surge control is “forward triage”—the sorting of patients before they arrive in the emergency department (ED). Direct-to-consumer (or on-demand) telemedicine, a 21st-century approach to forward triage that allows patients to be efficiently screened, is both patient-centered and conducive to self-quarantine, protecting patients, clinicians, and the community from exposure. It can allow physicians and patients to communicate 24/7, using smartphones or webcam-enabled computers [50].

This study has several limitations. It should be noted that the assessment of service quality was based on the opinions of patients and did not include the opinions of primary healthcare representatives, which would have provided a more complete picture of service quality. This study did not compare service quality before and after the COVID-19 pandemic, which would have allowed more useful lessons to be learned. Patients were recruited from public facilities, so it has a limited generalizability, as satisfaction rates in private facilities could have been much higher than those in public facilities due to the low caseload. Also, the introduction of compulsory health insurance began in Kazakhstan at the onset of the pandemic, which may have also impacted patient satisfaction rates. Additionally, the use of a self-administered questionnaire, while cost-effective, may have introduced response bias. One of the limitations of this study is the dichotomization of the satisfaction scale, where multiple response categories were collapsed into a binary variable (“satisfied” vs. “not satisfied”). This approach facilitates interpretation and statistical modeling; however, it may result in a loss of information regarding the nuances of satisfaction perception. Future research is encouraged to explore alternative coding approaches to ensure a more precise analysis. Another limitation of this study is the use of convenience sampling, which can introduce bias and limit the generalizability of results. Since participants are selected on the basis of their availability, not random selection, the selection may not fully reflect the wider population. This may affect how our results can be applied to other groups or settings. Although the results provide valuable information in the context of the sample population, caution should be exercised when extrapolating these results to larger or different populations. Future studies should aim to include a more diverse demographic sample and consider alternative data collection methods to minimize bias.

Finally, a key limitation is the absence of formal content and face validity assessments during the development of the questionnaire. While internal consistency and factor analysis confirmed the statistical reliability of the instrument, future research should prioritize comprehensive validation procedures to strengthen the construct validity of the survey tool.

Despite these limitations, important lessons for improving and enhancing the effectiveness of primary healthcare systems in the context of the COVID-19 pandemic can be drawn from the findings of this study. The data obtained during the study can help to develop strategies to improve the quality of services and the organization of primary healthcare in emergency situations. Assessing the role of medical examinations in influencing satisfaction levels can become the basis for reforms in preventive medicine. Therefore, it is obvious that there is a need to develop and implement quality management strategies at the PHC level during emergencies.

### Recommendations

Governments must invest significantly in infrastructure, capacity building, and strengthening primary healthcare services to ensure effective functioning during public health emergencies [51].

Improving communication between healthcare organizations is essential for timely emergency responses and process control [52]. Developing strategic guidelines for combating unknown infections, investing in primary healthcare infrastructure, and digitalizing domestic medicine are crucial. Special guidelines for managing patients with chronic diseases during emergencies should be developed, and automated systems for monitoring patient satisfaction should be implemented.

## 5. Conclusions

This study provides evidence on the factors influencing patient satisfaction with primary healthcare services in Almaty during the COVID-19 pandemic. Both demographic characteristics and facility-level attributes were found to significantly affect satisfaction levels. These findings underscore the need for targeted structural and organizational improvements in primary healthcare settings, especially during public health emergencies. Addressing these gaps through infrastructural upgrades, enhanced preparedness, and the integration of patient-centered care models can help to bolster trust and resilience within Kazakhstan’s healthcare system. Further research could include assessing the levels of satisfaction in public and private primary healthcare organizations and investigate how the introduction of Kazakhstan’s compulsory health insurance system has affected the levels of patient satisfaction in primary healthcare organizations.

## Figures and Tables

**Table 1:** Description of the study participants’ demographic and facility characteristics.

Variable	Satisfied N (%) or Mean ± SD	Rather Satisfied than Dissatisfied N (%) or Mean ± SD	Not Fully Satisfied N (%) or Mean ± SD	Rather Dissatisfied than Satisfied N (%) or Mean ± SD	Not Satisfied N (%) or Mean ± SD	Difficult to Answer N (%) or Mean ± SD
**Demographic Variables**						
Age	36.5 ± 14.3	31.2 ± 11.8	36.5 ± 13.1	37.0 ± 13.6	42.4 ± 13.2	39.8 ± 12.7
Gender						
Female Male	294 (28.4) 174 (16.8)	76 (7.3) 56 (5.4)	103 (10.0) 71 (6.9)	44 (4.3) 28 (2.7)	49 (4.7) 26 (2.5)	66 (6.4) 48 (4.6)
Marital status						
Married Single Widowed Divorced	213 (20.6) 159 (15.4) 30 (2.9) 66 (6.4)	60 (5.8) 63 (6.1) 3 (0.3) 6 (0.6)	96 (9.3) 57 (5.5) 6 (0.6) 15 (1.4)	33 (3.2) 30 (2.9) 3 (0.3) 6 (0.6)	45 (4.3) 21 (2.0) 3 (0.3) 6 (0.6)	48 (4.6) 36 (3.5) 15 (1.4) 15 (1.4)
Social status						
Employed Not employed Other Retired Student	263 (25.4) 58 (5.6) 26 (2.5) 68 (6.6) 53 (5.1)	68 (6.6) 15 (1.4) 9 (0.9) 22 (2.1) 18 (1.7)	104 (10) 32 (3.1) 8 (0.8) 19 (1.8) 11 (1.1)	42 (4.1) 7 (0.7) 2 (0.2) 14 (1.4) 7 (0.7)	41 (4.0) 7 (0.7) 6 (0.6) 12 (1.2) 9 (0.9)	57 (5.5) 19 (1.8) 12 (1.2) 17 (1.6) 9 (0.9)
Almaty region						
Turksyb Alatau Almaly Auezov Bostandyk Zhetysu Medeu Nauryzbai	60 (5.8) 44 (4.3) 83 (8.0) 71 (6.9) 46 (4.4) 55 (5.3) 56 (5.4) 53 (5.1)	16 (1.5) 13 (1.3) 23 (2.2) 17 (1.6) 12 (1.2) 14 (1.4) 23 (2.2) 14 (1.4)	19 (1.8) 24 (2.3) 18 (1.7) 23 (2.2) 25 (2.4) 17 (1.6) 21 (2.0) 27 (2.6)	9 (0.9) 8 (0.8) 2 (0.2) 17 (1.6) 8 (0.8) 4 (0.4) 7 (0.7) 17 (1.6)	10 (1.0) 8 (0.8) 4 (0.4) 4 (0.4) 10 (1.0) 9 (0.9) 12 (1.2) 18 (1.7)	15 (1.4) 18 (1.7) 13 (1.3) 8 (0.8) 14 (1.4) 16 (1.5) 22 (2.1) 8 (0.8)
Self-described health status						
Good Very good Satisfactory Not satisfactory Bad	192 (18.6) 120 (11.6) 141 (13.6) 9 (0.9) 6 (0.6)	57 (5.5) 21 (2.0) 48 (4.6) 6 (0.6) 0 (0.0)	72 (7.0) 24 (2.3) 66 (6.4) 12 (1.2) 0 (0.0)	27 (2.6) 9 (0.9) 30 (2.9) 3 (0.3) 3 (0.3)	36 (3.5) 6 (0.6) 27 (2.6) 6 (0.6) 0 (0.0)	51 (4.9) 15 (1.4) 45 (4.3) 3 (0.3) 0 (0.0)
Chronic disease						
No Yes	339 (32.8) 129 (12.5)	87 (8.4) 45 (4.3)	105 (10.1) 69 (6.7)	39 (3.8) 33 (3.2)	39 (3.8) 36 (3.5)	87 (8.4) 27 (2.6)
Dispanserization						
No Yes	228 (33.3) 78 (11.4)	66 (9.6) 12 (1.8)	81 (11.8) 36 (5.3)	48 (7.0) 3 (0.4)	42 (6.1) 21 (3.1)	63 (9.2) 6 (0.9)
Online consultation						
No Yes	84 (13.1) 204 (31.8)	30 (4.7) 45 (7.0)	54 (8.4) 51 (7.9)	33 (5.1) 15 (2.3)	54 (8.4) 6 (0.9)	36 (5.6) 30 (4.7)
**Facility characteristics**						
Entry and exit						
Yes No	387 (39.3) 60 (6.1)	87 (8.8) 33 (3.4)	108 (11.0) 57 (5.8)	51 (5.2) 18 (1.8)	54 (5.5) 18 (1.8)	90 (9.1) 21 (2.1)
Cross-ventilation system						
Yes No	393 (44.6) 39 (4.4)	93 (10.5) 24 (2.7)	99 (11.2) 42 (4.8)	21 (2.4) 33 (3.7)	18 (2.0) 39 (4.4)	63 (7.1) 18 (2.0)
Hand washing/sanitizing points						
Yes No	429 (43.3) 27 (2.7)	123 (12.4) 3 (0.3)	144 (14.5) 30 (3.0)	48 (4.8) 15 (1.5)	51 (5.2) 21 (2.1)	93 (9.4) 6 (0.6)
Face mask availability						
Yes No	306 (30.8) 144 (14.5)	66 (6.6) 57 (5.7)	81 (8.2) 87 (8.8)	21 (2.1) 48 (4.8)	12 (1.2) 60 (6.0)	60 (6.0) 51 (5.1)
Prophylactic measures						
Yes No	378 (40.8) 66 (7.1)	105 (11.3) 12 (1.3)	99 (10.7) 54 (5.8)	39 (4.2) 24 (2.6)	18 (1.9) 42 (4.5)	72 (7.8) 18 (1.9)
Assessment of sanitary and hygienic conditions						
Not satisfactory Satisfactory	6 (0.6) 459 (45.3)	0 (0.0) 129 (12.7)	18 (1.8) 153 (15.1)	21 (2.1) 45 (4.4)	21 (2.1) 51 (5.0)	9 (0.9) 102 (10.1)

Abbreviations: SD—standard deviation.

**Table 2:** Ordinal logistic regression of satisfaction with quality of medical services with demographic variables.

Variable	AOR	95% CI	*p*-Value
Demographic Variables			
Age	0.99	0.98–1.01	0.663
Gender			
Female Male	1 0.67	0.42–1.06	0.090
Marital status			
Married Single Widowed Divorced	1 0.93 1.04 2.32	0.59–1.49 0.46–2.37 1.36–4.05	0.780 0.928 **0.003**
Social status			
Employed Not employed Other Retired Student	1 0.33 1.53 2.02 1.25	0.18–0.60 0.75–3.20 1.07–3.89 0.70–2.26	**<0.001** 0.247 0.031 0.447
Almaty region			
Turksyb Alatau Almaly Auezov Bostandyk Medeu Nauryzbai Zhetysu	1 0.95 1.97 1.87 1.12 0.41 0.78 1.03	0.46–1.96 0.99–3.97 0.88–4.07 0.56–2.26 0.19–0.85 0.42–1.45 0.52–2.04	0.883 0.054 0.108 0.751 0.017 0.437 0.922
Self-described health status			
Good Bad Not satisfactory Satisfactory Very good	1 5.50 0.30 0.87 1.89	1.37–3.08 0.13–0.66 0.58–1.30 1.13–3.20	0.023 **0.003** 0.511 0.017
Chronic disease			
No Yes	1 0.76	0.51–1.15	0.198
Dispanserization			
No Yes	1 1.33	0.83–2.15	0.230
Online consultation			
No Yes	1 2.41	1.70–3.42	**<0.001**

Abbreviations: AOR—adjusted odds ratio; CI—confidence interval. Bold represents significant values.

**Table 3:** Ordinal logistic regression of satisfaction with quality of medical services with facility characteristics.

Variable	AOR	95% CI	*p*-Value
Facility Characteristics			
Entry and exit			
No Yes	1 1.35	0.94–1.93	0.102
Cross-ventilation system			
No Yes	1 1.96	1.37–2.79	**<0.001**
Hand washing/sanitizing points			
No Yes	1 1.14	0.71–1.82	0.578
Face mask availability			
No Yes	1 1.48	1.11–1.98	0.007
Prophylactic measures			
No Yes	1 1.39	0.96–2.01	0.076
Assessment of sanitary and hygienic conditions			
Not satisfactory Satisfactory	1 2.04	1.22–3.42	0.006

Abbreviations: AOR—adjusted odds ratio; CI—confidence interval. Bold represents significant values.

## Data Availability

The data that support the findings of this study are available on request from the corresponding author.
